# Errata

**Published:** 1897-02

**Authors:** 


**Errata.**—In our January number, the name of Prof. Smith appeared as "Albert J. Smith." It should have been "Allen J. Smith." On page 404, first word of third line from the bottom, for "fort" read "*forte.*"

